# Parenting in the Digital Era: Quantitative and Qualitative Insights from Families of Children with Neurodevelopmental Disorders

**DOI:** 10.3390/children13060716

**Published:** 2026-05-22

**Authors:** Niccolò Butti, Eleonora Mascheroni, Vittoria Maucci, Roberta Nossa, Lucia Scaccia, Francesca Masserano, Emilia Biffi, Rosario Montirosso

**Affiliations:** 1 Scientific Institute, IRCCS E. Medea, 0-3 Centre for the At-Risk Infant, Bosisio Parini, Lecco, Italy; niccolo.butti@lanostrafamiglia.it (N.B.); rosario.montirosso@lanostrafamiglia.it (R.M.); 2 Scientific Institute, IRCCS E. Medea, Bioengineering Lab, Bosisio Parini, Lecco, Italy; roberta.nossa@lanostrafamiglia.it (R.N.); emilia.biffi@lanostrafamiglia.it (E.B.)

**Keywords:** digital media use, neurodevelopmental disorders, parents’ perspectives, digital parenting, thematic analysis

## Abstract

**Highlights:**

**What are the main findings?**

- Parents of children with neurodevelopmental disorders show divergent attitudes, with broadly similar proportions reporting positive, neutral, and negative views.
- More positive perceptions are associated with greater child disability severity and with viewing digital media as supportive for parenting.

**What are the implications of the main findings?**

- Family-centred guidance is needed to support parents of children with neurodevelopmental disorders in managing screen use, while reducing stress and improving regulatory strategies.
- Assistive and inclusive digital approaches should be promoted to enhance participation and developmental opportunities for children with neurodevelopmental disorders.

**Abstract:**

Background/Objectives: This study explored parents’ perspectives regarding digital media use in children and adolescents with neurodevelopmental disorders (NDs) and examined how these views vary according to family and clinical characteristics. Methods: Data were collected from an Italian survey involving 352 families. Items assessed the perceived effects of digital devices on child development and parenting, awareness of screen time guidelines, and use of time- and content-limiting tools. Quantitative analyses were complemented by a reflexive thematic analysis of open-ended responses describing how digital media influenced parenting. Results: Parents expressed divergent attitudes towards digital media, with broadly similar proportions reporting positive, neutral, and negative views regarding both child development and parenting. More favourable views were associated with greater perceived benefits for children and were more frequent among parents of children with more severe functional disabilities. About half had discussed screen use with health professionals, and most were aware of existing guidelines. Thematic analysis identified six themes related to digital parenting: *educational means* (digital devices as tools for communication, learning, and socialisation), *entertainment* (screens as a source of leisure or behavioural management), *reward* (digital media used as reinforcement), *screen time as a “necessity”* (technology as an integral and sometimes rehabilitative part of daily life), *negative effects on the child* (concerns about detachment, reduced social interaction, and mood dysregulation), and *parental behaviour and attitudes* (reflecting the emotional burden of regulation and broader beliefs about digital media). Conclusions: Parents of children with NDs navigate digital media use through a complex balance of perceived risks and benefits. Findings highlight the need for family-centred guidance and assistive technology approaches that promote digital inclusion while addressing parental stress and regulatory challenges.

## 1. Introduction

Neurodevelopmental disorders (NDs) include conditions with different aetiologies and clinical presentations, such as intellectual disability, genetic syndromes, Autism Spectrum Disorder (ASD), learning disabilities, and Attention Deficit and Hyperactivity Disorder (ADHD) [1]. NDs emerge during the developmental period and are characterised by challenges in multiple areas and frequent comorbidity, often leading to significant restrictions in autonomy and daily living activities [2]. For individuals with NDs, the ability to navigate an increasingly digital world has become an essential skill for full participation in contemporary society. The concept of inclusion has accordingly expanded to encompass digital citizenship, defined as the possibility to access and use digital media in an ethical, safe, and responsible way [3].

At the same time, a growing body of research has documented the potential adverse effects of excessive screen exposure on child development and health, underscoring the risks associated with digital media use among both typically developing children and those with NDs [4,5,6]. Most studies on clinical samples have focused on ASD, suggesting that this population tends to have longer daily screen exposure and is at a higher risk of screen addiction [7,8]. Screen time in ASD has thus been seen as both an environmental risk factor and a behavioural response to neurobiological vulnerabilities [9,10]. Building on the general screen time recommendations issued by paediatric societies across different countries [11,12,13,14,15], recent efforts have sought to develop guidelines for children and youth with ASD, which promote digital citizenship while mitigating risks [16,17]. Nevertheless, evidence from both typically developing and ND populations indicates that only a minority of children meet these recommendations [18,19]. The relationship between digital media and child development in NDs is therefore best understood not as a simple dichotomy between risk and benefit, but as a dynamic tension shaped by the type and context of use, the child’s specific profile of strengths and vulnerabilities, and the surrounding family and social environment. Digital media can serve as a meaningful tool for learning, communication, and participation [20,21], while simultaneously posing risks of overuse, social withdrawal, and behavioural dysregulation [22,23].

Given these challenges, understanding how children and adolescents navigate the digital ecosystems becomes crucial. To address this, recent technical and policy reports from the American Academy of Pediatrics have contextualised children’s digital media experiences by adopting Bronfenbrenner’s socioecological model [24]. Within this framework, caregivers’ attitudes and behaviours towards digital media play a pivotal role in shaping children’s screen habits [25,26]. This also implies that the burden of finding a balance between children’s participation in a digital society and the potential risks of excessive screen exposure largely falls on parents [27]. Managing and supporting children’s engagement with digital devices has therefore become a central feature of modern parenting [28]. From this perspective, the strategies and behaviours that parents adopt to regulate the amount, content and context of screen time have been described as digital parenting or media parenting [29,30]. These practices are influenced by multiple factors, including socioeconomic background, the caregiver-child relationship, parenting stress, and caregivers’ own media use and approaches towards technology [25,31]. In the case of children with NDs, media parenting may be further shaped by unique characteristics and challenges, such as functional impairments and frequent comorbidities, which can limit children’s access to and independent use of digital devices [30,32].

However, research examining media parenting in families of children with NDs remains limited and has yielded mixed findings. For instance, Alfredsson Ågren and colleagues reported that fewer parents of adolescents with intellectual disability had concerns about online risks for their adolescents compared with parents of typically developing peers [33]. Similarly, a qualitative study based on focus groups with parents of children with intellectual disability found that internet use was often viewed as a valuable tool for social connection and peer interaction [34]. In contrast, a recent study conducted in Turkey showed that parents of children with NDs perceived social media as largely non-beneficial and expressed strong concerns about their use [31]. Notably, more negative perceptions may lead to an increased reliance on restrictive media strategies [35], potentially limiting opportunities for digital inclusion of children with NDs [36]. At the same time, families should be aware of the potential developmental and behavioural consequences of excessive screen exposure and implement effective regulation strategies. Existing tools, such as timers, auto-lock functions, and parental control apps, may help parents limit screen time and content. Beyond restrictive approaches, however, digital media have become an integral part of daily family life and can serve as a parenting resource fulfilling multiple functions, such as entertainment, education, or behaviour regulation [37]. Digital interventions targeting parents of children with NDs have shown promising evidence of effectiveness, and may be particularly valuable in contexts where access to in-person services is limited [38,39]. Taken together, these considerations underscore the importance of investigating digital parenting among parents of children with NDs, and specifically, how parents perceive digital devices as either facilitating or hindering their parental role.

To address these underexplored issues, the present study adopts an exploratory approach, using a survey-based design to capture a broad range of parental perceptions, attitudes, and practices among families of children and adolescents with NDs. Given this design, the measures employed consist of single survey items, which are appropriate for descriptive and exploratory purposes but do not allow for the depth of assessment afforded by validated multi-item instruments. The overarching goal was to provide both quantitative and qualitative insights into how parents of children with NDs perceive, manage, and experience digital media use in everyday family life. Specifically, the study aimed to: a) examine parents’ perceptions and attitudes regarding the effects of digital media on child development and their influence on parenting; b) assess parental awareness of existing screen time guidelines, whether digital exposure was discussed with health professionals, and the use of time- and content-limiting tools; c) explore whether these perspectives varied according to family and clinical characteristics, such as disability severity or socioeconomic status (SES); and d) identify key themes describing the perceived effects of digital media use on parenting.

## 2. Materials and Methods

### 2.1. Procedure and Participants

The items analysed in this study were part of a wider survey distributed among families of children with NDs between 18 December 2024, and 26 February 2025 using REDCap (Research Electronic Data Capture) software, version 15.5.36 (Vanderbilt University, Nashville, TN, USA). Data from 352 Italian families, corresponding to a total of 407 children and adolescents with NDs, were collected in a completely anonymous, aggregated form. The sample reflected the diagnostic heterogeneity typical of paediatric populations referred to neurorehabilitation services; the most represented diagnoses were genetic syndromes (24%), ASD (16%), and learning disabilities (13%). A detailed description of procedures and the recruited sample is reported in a previously published paper focusing on child and parent screen time [19]. Participants provided informed consent by choosing to proceed with the online survey. The study protocol was reviewed and authorised by the institutional office for Ethics Committee and Clinical Studies of IRCCS E. Medea—La Nostra Famiglia, which also verified, in consultation with the competent territorial Ethics Committee (Comitato Etico Territoriale Lombardia 2), that formal ethical review was not required for this study design. In parallel, the Data Protection Officer (EU Regulation 2018/1725) of IRCCS E. Medea assessed and approved all procedures related to data handling and privacy compliance. All procedures were conducted in accordance with the Declaration of Helsinki and with applicable regulations for research involving human participants.

### 2.2. Measures

The published survey included multiple sections assessing sample demographics, clinical information for each child with NDs, child screen time, parent screen time and stress [19]. An additional section was developed to explore parents’ perspectives, awareness, and practices related to children’s screen use. Given that existing validated instruments were not specifically designed for children with NDs across a wide age range [40,41,42], the research team developed an ad hoc questionnaire through an iterative process informed by the existing literature [43,44] and by clinical expertise in NDs. A draft version was administered to approximately ten families of both typically developing children and children with NDs to verify the clarity and comprehensibility of the items. Feedback from this piloting phase was used to refine the wording and structure of the questions until consensus within the research team was reached on the final version. The items were therefore not designed as components of psychometric instruments intended to measure latent constructs, but rather as descriptive queries aimed at capturing parents’ stated perceptions, attitudes, and practices towards digital media use. This approach is consistent with the exploratory aims of the study and with methodological precedents in survey-based research on parental perspectives in this field [44,45]. It should be noted that other sections of the broader survey included validated multi-item scales assessing parental stress and difficulties in controlling children’s media exposure; satisfactory psychometric properties for these instruments were reported in the previously published study from the same dataset [19].

Two items employed a 5-point Likert scale to evaluate parents’ perceived impact of digital devices on their child’s development (item 1—*Benefits for child development*) and their perception of the effects of digital media use on their parental role (item 2—*Support for parenting*). For the latter, parents were further invited to provide an open-ended description of how digital device use facilitated or did not facilitate their parental role. Then, four dichotomous (“Yes/No”) items explored whether parents had discussed screen-related issues with health professionals (Item 3—*Discussed with health professionals*), were aware of existing screen time guidelines (Item 4—*Aware of guidelines)*, used timers or other time-limiting tools (Item 5—*Time-limiting tools*), and used parental controls or other content-limiting tools (Item 6—*Content-limiting tools*). The English translation of the items is presented in Table 1.

### 2.3. Data Analysis

#### 2.3.1. Quantitative Analyses

Descriptive analyses were performed for each survey item, with response frequencies expressed as percentages. Statistical analyses were carried out using SPSS29 for Windows, with the significance level set at *p* < 0.05. A first *chi-square* test was conducted to examine the association between parents’ perceived effect of digital devices on child development and their perception of digital media use in parenting. To ensure more balanced distributions across categories, responses to both items were recoded into three levels: “Yes”, combining “Many benefits”/”Very much” and “More benefits than negative consequences”/”Somewhat yes”; “Neutral”, corresponding to “Neither benefits nor negative consequences” and “Neither yes nor no”; “No”, combining “More negative consequences than benefits”/”Somewhat no” and “Many negative consequences”/”Not at all”. Then, each item was examined separately through *chi-square* tests according to family and clinical characteristics. As some families had more than one child with NDs (N children = 407), child-level variables such as child age range, biological sex, and main diagnosis were not included in these analyses, given that their assignment to a single-family unit would have been ambiguous. Instead, for families with more than one child with NDs, the highest level of functional disability among siblings was retained as the variable entered in the analyses. This approach was chosen to reflect the overall caregiving demands experienced by the family unit, which represented the primary level of analysis in the present study. Variables with very few responses in some categories were recoded and collapsed to ensure each category had adequate cell counts (>10%), in order to limit potential errors or inflated significance due to skewed distributions and multiple response levels. For instance, SES, assessed using the MacArthur Scale of Subjective Social Status [46], was recoded as “Low/middle” (scores 1–5), “Middle-high” (scores 6–7), and “High” (scores 8–10). Only mothers and fathers were considered as responding caregivers, given that the “Other” category comprised only two participants.

Given the exploratory nature of the present study and the absence of a priori directional hypotheses, no correction for multiple comparisons was applied. This approach is consistent with methodological recommendations for exploratory research, in which Type I error correction procedures such as Bonferroni are considered overly conservative and risk increasing Type II error by obscuring potentially meaningful patterns relevant to future investigation [47,48]. Findings involving small effect sizes or borderline significance levels should accordingly be interpreted with caution and treated as tentative rather than conclusive.

#### 2.3.2. Qualitative Analyses

The open-ended responses at item 2.1 were first screened to exclude uninformative answers. *Chi-square* tests were performed to verify whether the subgroup of parents who provided open-ended responses differed from non-respondents in their socio-demographic or clinical characteristics. The responses were then analysed using a reflexive thematic analysis approach [49,50], following six phases: (1) familiarization with the data, (2) generation of initial codes, (3) identification of preliminary themes, (4) review of themes, (5) definition and naming of themes, and (6) production of the analytic report. Adopting a hybrid inductive–deductive approach [51,52], data-driven themes were iteratively compared and refined in light of the thematic structure proposed by a recent systematic review and meta-synthesis on children’s screen time [53]. Consistent with this iterative, interpretative process, although informed by previous research, the themes identified did not exactly replicate those reported in Chong et al. (2023) [53].

The research team comprised developmental and clinical psychologists with extensive expertise in NDs, both in terms of neuropsychological profiling and parent support intervention, working within a paediatric neurorehabilitation institute in Northern Italy where clinical practice is grounded in a family-oriented approach to assessment and intervention. The broader team also included two biomedical engineers (RN, EB) specialising in robotics and assistive technologies. This interdisciplinary background inevitably shaped the researchers’ sensitivity to themes related to parenting burden, assistive technology, and the tension between clinical guidance and everyday family life. These orientations were made explicit during the analytic process and treated as resources for interpretation rather than as sources of bias to be eliminated.

Two researchers (NB, FM) independently engaged with the data in the initial coding phase, generating codes through close reading of the responses, to enrich data interpretation rather than to reach consensus on meaning [54]. Codes and emerging themes were then discussed iteratively between the two coders, with analytic decisions documented through a reflective log. Where interpretive differences arose, these were resolved through discussion and, where needed, by returning to the data. A third researcher (VM) contributed to the review, refinement, and naming of themes and subthemes, providing an additional layer of critical reflection on the analytic process. The final thematic structure was then presented to and discussed with the full research team, whose interdisciplinary perspectives contributed to the interpretive consolidation of the themes and ensured that their meaning was examined from multiple professional standpoints. Themes were conceptualised as patterns of shared meaning actively co-constructed through the interaction of data, analytic reflection, and researcher subjectivity [50].

Regarding thematic completeness, the concept of data saturation as traditionally operationalised in grounded theory or interview-based research is not directly applicable to thematic analysis conducted on a fixed survey dataset, where the corpus is defined at the outset and cannot be iteratively expanded. In this context, saturation cannot be pursued procedurally in the conventional sense, and its application to thematic analysis more broadly remains a debated and evolving topic in qualitative methodology [55]. Consistent with recent methodological guidance, thematic completeness was therefore approached conceptually: themes were identified and retained even when present in only a small number of responses, in order to remain sensitive to patterns that might diverge from the existing literature, particularly given that the meta-synthesis focused exclusively on typically developing children [53].

## 3. Results

A description of the sample according to demographic and clinical variables used in the analysis is provided in Table 2.

### 3.1. Quantitative Analyses

Answers about parents’ perspectives, awareness, and practices regarding child screen time are summarised in Table 3.

While a portion of parents perceived screens as beneficial for children or as helpful tools for their parental role (39% and 26%, respectively), others viewed them as neutral (26%; 32%) or potentially detrimental (35% and 42%). Overall, a more favourable perception of digital screens for parenting was associated with a more positive appraisal of their impact on children (χ^2^ = 83.39, *p* < 0.001, Cramer’s *V* = 0.48). Specifically, parents who perceived screens as supportive tools in their parental role were more likely to evaluate their effects on children positively (71%), whereas those who viewed screens as interfering with their parental role tended to report negative consequences for their children (55%). Half of the respondents stated having discussed screen-related issues with health professionals, and most were aware of existing guidelines on children’s media use. The use of content-limiting tools was confirmed in about half of the sample, while time-limiting tools were relatively less common.

Table 4 reports parents’ perspectives about child screen time according to demographic and clinical characteristics of the sample.

Fathers showed a more favourable attitude towards digital devices than mothers, with approximately half reporting that digital media offer more benefits than drawbacks for children’s development (χ^2^ = 10.96, *p* = 0.004, Cramer’s *V* = 0.18) and acknowledging them as helpful tools in their parental role (χ^2^ = 9.76, *p* = 0.008, Cramer’s *V* = 0.17). No other significant respondent-related differences emerged (all χ^2^ < 0.12, all *p*s > 0.730). A higher use of time-limiting tools was observed among younger compared with older parents (χ^2^ = 4.53, *p* = 0.033, Cramer’s *V* = 0.11), whereas all other comparisons were non-significant (all χ^2^ < 4.13, all *p*s > 0.126). No significant differences emerged for SES, although there was a trend towards significance for use of time-limiting tools (χ^2^ = 5.99, *p* = 0.050, Cramer’s *V* = 0.13; all other χ^2^ < 3.36, all other *p*s > 0.224). The number of children with NDs in the family did not significantly affect parents’ responses (all χ^2^ < 2.47, all *p*s > 0.115). Conversely, significant differences emerged across levels of functional disability, with the exception of guideline awareness (χ^2^ = 1.58, *p* = 0.453, Cramer’s *V* = 0.07). More severe child disabilities were associated with more favourable attitudes towards the effects of digital media use on child development (χ^2^ = 27.05, *p* < 0.001, Cramer’s *V* = 0.28) and parental role (χ^2^ = 23.45, *p* < 0.001, Cramer’s *V* = 0.26). Parents of children with moderate or severe-to-complete disabilities more frequently reported having discussed screen exposure with health professionals (χ^2^ = 6.63, *p* = 0.036, Cramer’s *V* = 0.14), whereas a lower proportion of parents of children with severe-to-complete disabilities declared using time-limiting tools (χ^2^ = 7.89, *p* = 0.019, Cramer’s *V* = 0.15). A similar trend, approaching significance (χ^2^ = 5.94, *p* = 0.051, Cramer’s *V* = 0.13), was observed for the use of content-limiting tools.

### 3.2. Qualitative Analyses

Open-ended responses regarding how the use of digital devices supported or did not support parents in their parental role were provided by 244 families. Seventeen responses were excluded as uninformative (e.g., “I don’t know”, “Nothing to add”). Therefore, thematic analysis was conducted on 227 valid responses (76 “Yes”, 68 “Neutral”, 83 “No”). Preliminary *chi-square* analyses comparing this subgroup with non-responding families revealed a significant difference for responding parents, as fathers were more likely (79%) than mothers (61%) to provide a response (χ^2^ = 6.77, *p* = 0.034, Cramer’s *V* = 0.14). A trend approaching significance (χ^2^ = 3.74, *p* = 0.053, Cramer’s *V* = 0.1) emerged for the number of children with NDs, indicating that families with more than one affected child were more likely to respond (76%) than those with a single child with NDs (62%). These differences partly balanced the composition of the overall sample, in which mothers with only one child with NDs were more prevalent. No other socio-demographic and clinical differences were found between responding and non-responding families (all χ^2^ < 1.26, all *p*s > 0.263).

The analysis identified six overarching themes and twelve subthemes, which are described in detail in the following sections. It is worth noting that some parents mentioned more than one theme or subtheme in their responses, resulting in a total of 308 coded excerpts. The themes reflected a spectrum of parental orientations towards digital media, ranging from actively positive, where screens were embraced as educational or rehabilitative tools, to pragmatically neutral, where digital media were acknowledged as an inevitable feature of modern life, to predominantly negative, where concerns about developmental harm and parental authority predominated. Several themes were conceptually interrelated. For instance, the *educational means* and *screen time as a “necessity”* themes both reflected an instrumental and often needs-driven relationship with digital media, particularly evident among parents of children with more severe functional limitations, whereas the *entertainment* and *reward* themes captured a more situational use of screens as a coping or management strategy within the demands of daily caregiving. The *negative effects on the child* and *parental behaviour and attitudes* themes were similarly interconnected, as parental concerns about child outcomes frequently co-occurred with reflections on the difficulty of regulating screen use and the emotional burden this imposes. The results of the thematic analysis are summarised in Table 5.

#### 3.2.1. Educational Means

This theme, consistent with that reported in Chong et al. [53], reflected parents’ recognition of digital media as a support for both themselves and their children. It was primarily endorsed by parents who perceived digital devices as facilitating their parental role, although this was also identified among neutral or negative answers. Parents recognised digital media as a critical support for *communication*, particularly among children with severe-to-complete functional disabilities (*n* = 11). Similarly, digital devices were perceived as a *learning* aid, fostering attention, concentration, autonomy, and broader developmental progress. The *educational means* theme also encompassed *socialisation*, such as using digital media for shared parent–child activities or to sustain peer relationships, and *activities for parents*, referring to online resources and materials that helped parents support their children’s development. Notably, the *socialisation* subtheme recurred in the *negative effects on the child* theme, where digital devices were instead perceived as a barrier to social interaction. This conceptual tension reflected the dual and context-dependent nature of digital media use in this population.

#### 3.2.2. Entertainment

This theme included responses describing digital devices as a means for entertaining children. The *leisure time* subtheme captured parents’ perception of digital media as a source of relaxation and enjoyment during free time. Conversely, the *digital pacifier or babysitter* subtheme referred to the use of screens to keep children calm or occupied while parents attended to other tasks, reflecting a more instrumental and need-driven function that, while common, sat in tension with current screen time guidelines.

#### 3.2.3. Reward

Although this theme appeared less frequently than others, some parents described using digital devices as a *reward* or reinforcement strategy to manage behaviour, particularly in children with oppositional or defiant tendencies. This use was conceptually distinct from *entertainment*, in that it involved a deliberate regulatory function rather than simple engagement and partly overlapped with the *parental control and authority* subtheme in reflecting the challenges parents face when managing their children’s behaviour in everyday contexts.

#### 3.2.4. Screen Time as a “Necessity”

In line with [53], some parents acknowledged digital media as an integral part of everyday life and a “necessity” in the modern world, reflecting a pragmatic and largely non-evaluative stance. This theme also included responses describing the necessity of using facilitative or rehabilitative digital devices (e.g., electronic communication aids for non-verbal children, learning tools for children with specific learning disorders), often under clinical guidance. In this respect, the *necessity* theme was closely related to the *educational means* theme but differed in emphasis: whereas *educational means* focused on the perceived benefits of digital media for development and learning, *necessity* framed screen use as an unavoidable or clinically indicated reality, irrespective of its positive or negative valence.

#### 3.2.5. Negative Effects on the Child

Parents frequently reported negative effects of screen time on their children. While partly overlapping with the results of Chong et al. [53], this theme focused specifically on perceived actual effects rather than general attitudes towards screen exposure. Parents often described children as becoming *detached from reality* (e.g., over-absorbed, isolated, or confused between real and virtual experiences), a subtheme that stood in direct contrast to the engagement and learning opportunities described in the *educational means* theme. They also reported that digital media use reduced opportunities for *socialisation* and dialogue with parents and peers, echoing the *socialisation* subtheme identified within educational means but with an opposite valence. The *mood and behaviour* subtheme encompassed aggressive or oppositional behaviours, particularly when children were asked to interrupt screen time, as well as inappropriate language, irritability, mood changes, and increased anxiety linked to screen content. This subtheme was often connected to the *parental control and authority* subtheme, as behavioural difficulties during or after screen use were frequently cited as a source of parenting strain and conflict.

#### 3.2.6. Parental Behaviour and Attitudes

This theme captured parents’ reflections on their own behaviours, attitudes, and beliefs regarding digital media use and its effects on their parental role and functioned as a meta-level theme that cut across and contextualised many of the others. The *parental control and authority* subtheme included reports of strain associated with regulating children’s screen use, such as guilt, conflict, difficulty in setting limits, and a perceived weakening of parental authority. This subtheme was closely linked to the *negative effects on the child* theme, as concerns about harm frequently underpinned the difficulties in regulation. The *beliefs and values* subtheme reflected parents’ broader conceptions of parenting and digital media, predominantly negative and extending beyond their specific experiences with their own children, often shaped by wider social and cultural discourses about screen time. Lastly, a more neutral position recognised digital media as *“just a tool”*, whose impact depended on conscious, supervised, and balanced use, without a priori attributions of positive or negative value. This stance resonated with the *necessity* theme in its pragmatic, non-evaluative framing, and implicitly challenged the moral loading present in the *beliefs and values* subtheme.

## 4. Discussion

This survey-based study explored parents’ perspectives, awareness, and practices related to screen use in children and adolescents with NDs. Quantitative analyses examined responses according to family and clinical characteristics, while a thematic analysis was conducted to capture parents’ descriptions of how digital media use influenced their parental role. Given the exploratory nature of the study, the following findings should be interpreted descriptively, as patterns warranting further investigation rather than as definitive conclusions. Overall, parents expressed heterogeneous views regarding the developmental impact of digital devices and their effects on their parental role, with relatively similar percentages of the sample showing either negative, neutral, or positive attitudes. These results mirror the conflicting and mixed findings of previous studies exploring parents’ perception of specific aspects of digital media use among individuals with NDs, such as social media [31,56] and internet access [33,36]. Such variability may also reflect the broader challenge of balancing digital media use in everyday life with the morally-charged social discourses about screen time [57].

As expected, parents who reported a more favourable perception of screen use in facilitating their parental role also tended to perceive greater benefits of digital media for their children, an association of medium-to-large magnitude. Considering clinical and family variables in the analysis provided further insights into this association. Specifically, parents of children with more severe functional disabilities expressed more positive views towards digital media use, in terms of both its developmental impact on the child and its supportive function in parenting, both representing small-to-medium effects. More than half of these parents also reported having discussed screen exposure with health professionals. These associations are consistent with the hypothesis that, in more severe conditions, digital devices may serve critical rehabilitative or assistive functions, such as augmentative and alternative communication tools for non-verbal children [58], and that functional limitations may restrict the range of activities available to children with severe disabilities [59], making screen-based activities among the few that allow them a sense of autonomy and engagement. At the same time, digital media may offer caregivers temporary relief from the particularly demanding aspects of raising a child with severe disabilities [60,61,62], which is consistent with previous findings showing an association between parental stress and child screen time in this population [19]. However, these interpretations remain speculative in the context of the present cross-sectional, survey-based design, and should be treated as hypotheses to be examined in future research with more targeted methodologies.

Fathers showed a more favourable attitude towards digital devices than mothers, with approximately half reporting that digital media offer more benefits than drawbacks for children’s development and acknowledging them as helpful tools in their parental role. These differences, while statistically significant, were small in magnitude and should be interpreted with caution. The direction of these associations is consistent with previous research on typically developing populations documenting similar gender differences [63,64], and with findings suggesting that fathers may hold more favourable attitudes towards technology [65] and be less likely to set restrictions on child screen time [53]. However, given the exploratory and cross-sectional nature of the present study, these findings should not be interpreted causally. The observed differences may reflect a range of unmeasured factors, including differential exposure to caregiving demands, divergent information-seeking behaviours, or cultural norms around parental roles and technology use. These associations warrant further investigation in studies specifically designed to examine paternal attitudes and involvement in digital parenting for children with NDs.

Most parents were aware of existing guidelines on screen time, regardless of family and clinical variables. Approximately half reported using content-limiting tools, while time-limiting tools were relatively less common. The latter were more frequently used by younger parents, a statistically significant though small effect. Younger parents may have stronger digital skills and, consequently, greater confidence in their management of child screen time [30,66]. This finding may also reflect that younger parents likely had younger children, since time-limit recommendations are typically targeted at early developmental stages [11,13]. Although no significant differences emerged for SES, a trend towards significance was observed for time-limiting tools, with higher-SES families showing greater use of such strategies. This pattern is consistent with mixed findings in the literature on typically developing children, where associations between SES and digital parenting practices have been reported in some studies but not others [67,68], suggesting that socioeconomic and cultural factors may shape parental regulatory behaviours in ways that warrant further investigation. Conversely, a reduced use of both time- and content-limiting tools was observed among parents of children with more severe functional disabilities, likely due to the fact that these children are less autonomous in using digital screens and more frequently require the presence of a caregiver [19].

The qualitative findings from the thematic analysis both corroborate and enrich the quantitative results, revealing the experiential texture underlying the statistical patterns. For instance, the more favourable views observed among parents of children with more severe disabilities find a direct echo in the *educational means* and *screen time as a “necessity”* themes, within which parents explicitly described digital devices as indispensable communication and learning tools for children with significant functional limitations [21,69,70]. Similarly, the association between positive parenting perceptions and higher disability severity resonates with the *entertainment* and *reward* themes, where digital media emerged as a practical coping strategy within demanding caregiving contexts. Conversely, the predominantly negative attitudes expressed by a substantial proportion of parents are reflected in the *negative effects on the child* and *parental behaviour and attitudes* themes, which capture not only concerns about developmental impact but also the emotional burden of regulating screen use.

Although existing guidelines advise against screen time as a *digital pacifier or babysitter* [13,71], this practice was clearly described in many parental responses. Considering that most parents reported being aware of current guidelines, and that this usage was found only in a minority of children in the previous study on the same population [19], this finding needs further elaboration. Inconsistencies between parents’ attitudes towards digital media, their children’s screen time, and actual parenting practices have been widely documented [27]. These apparent contradictions have been interpreted within the uses and gratification theory framework, which posits that screen time can satisfy a range of parent needs, such as occupying the child, calming them, or rewarding desired behaviour [37,72]. At the same time, digital parenting is strongly influenced by prevailing cultural and social conceptions of what a ‘good parent’ should do and by the often negative and contradictory discourses about screen time [27,57]. This moral framing emerged in the *beliefs and values* subtheme, where many parents attributed an a priori negative value to digital media use in parenting. The pressure to conform to normative ideals may heighten the emotional strain, contributing to feelings of guilt, frustration, and a perceived loss of authority [45,73], as reflected by the responses clustered in the *parental control and authority* subtheme. Conversely, parents who expressed a more neutral stance described digital devices as *“just a tool”* in their children’s daily life and acknowledged access to digital media as a *“necessity”* in the contemporary world [53]. This sense of necessity may be particularly perceived among families of children with NDs, for whom digital technologies can address specific rehabilitative or assistive needs.

Nonetheless, concerns were also expressed about excessive absorption, reduced social interaction, and negative effects on mood and behaviour. These problems, widely reported in typically developing children [53], may be amplified in children with NDs due to cognitive and emotional-behavioural vulnerabilities [32]. Interestingly, digital devices were perceived both as facilitators and as barriers to *socialisation* [31,74], a subtheme detected across both the *educational means* and *negative effects on the child* themes. Overall, these findings highlight the need to support families with NDs in digital parenting [30], not only to monitor and mitigate risks but also to promote digital inclusion of children with NDs as a part of their broader right to social participation [3,16]. In this regard, a ‘positive risk-taking’ approach has been recommended to balance the potential risks of digital media use with the benefits of digital participation and empowerment for individuals with NDs [33,36,75].

The present study was designed as an exploratory investigation; accordingly, key variables were assessed using single survey items, which are appropriate for this purpose but limit the depth and precision with which the constructs of interest can be captured. Future studies should consider more comprehensive and validated measurement approaches to examine these constructs in greater depth [40,41]. Conducting direct, in-depth, or semi-structured interviews would also allow for a richer and more nuanced understanding of parents’ perspectives on digital media use in children with NDs. Findings should therefore be interpreted as indicative thematic patterns rather than as the outcome of in-depth qualitative inquiry, and the level of interpretive claims has been calibrated accordingly. Not all participants provided responses to the open-ended question; preliminary analyses confirmed that respondents and non-respondents differed only with respect to the proportion of fathers versus mothers, and showed a trend for families with more than one child with NDs, both differences being small in magnitude, which limits but does not substantially undermine the generalisability of the qualitative findings. Moreover, only parents’ reports were considered, without incorporating children’s own experiences or perceptions; this represents an important limitation, as perspectives on digital media use can differ substantially between individuals with disabilities and their parents or caregivers [76]. Second, as the survey was distributed anonymously and partly through family associations and clinical networks, self-selection bias cannot be excluded. Families who participated may have been more engaged with or concerned about digital media issues than non-respondents, which could affect the generalisability of findings. The anonymous online design also precluded calculation of a formal participation rate. At the same time, the heterogeneity of attitudes observed and the consistency of the identified themes with prior literature provide some reassurance regarding the breadth of perspectives captured. Finally, the sample was composed of families of children with a wide range of NDs, reflecting the heterogeneous population typically referred to paediatric neurorehabilitation services. While this ecological representativeness is a strength of the study, the variability across diagnostic groups, together with the small and unequal sample sizes within each condition, limited the generalisability of the results and the possibility of conducting condition-specific analyses (e.g., for ASD or cerebral palsy separately). The use of the highest functional disability level among siblings as a proxy variable, while justified by the family-level unit of analysis, further constrains the interpretability of findings at the level of individual diagnoses. These considerations should be borne in mind when interpreting the present results and designing future studies with larger, diagnosis-specific samples.

## 5. Conclusions

Despite these limitations, this study provides exploratory evidence on how families of children with NDs navigate the complex landscape of digital media use, integrating quantitative and qualitative perspectives to capture both the breadth and the experiential texture of parental attitudes. The results suggest that digital devices can serve as meaningful educational and rehabilitative tools for children with NDs, while also posing potential risks for overuse and reduced interaction. These findings, interpreted cautiously given the exploratory design and the methodological constraints described above, point to several specific directions for clinical practice. Family-centred guidance on digital media use should be routinely integrated into paediatric neurorehabilitation services, with content tailored to the child’s disability level. This guidance should address not only the risks of excessive screen exposure but also the potential of digital tools as assistive and rehabilitative resources for children with NDs, in line with their broader right to digital citizenship. Parents should be supported in developing balanced regulatory strategies that account for individual family needs, and the emotional burden associated with digital parenting should be explicitly acknowledged in the context of clinical support.

Future research should examine parental digital attitudes in larger, diagnosis-specific samples using longitudinal designs, validated multi-item instruments, and richer qualitative methods such as in-depth interviews or focus groups. Such approaches would allow researchers to move beyond exploratory description towards a more robust understanding of the mechanisms underlying these patterns and their impact on child outcomes.

## Figures and Tables

**Table 1:** English translation of survey items exploring parents’ perspectives, awareness, and practices about child screen use.

Item	Question	Response Format
1	Regarding your child’s development (cognitive, language, social, etc.), do you think that the use of digital devices has…	5-point Likert scale ranging from Many benefits to Many negative consequences
2	Do you think that the use of digital devices may facilitate your parental role?	5-point Likert scale ranging from Very much to Not at all
2.1	Please briefly describe how the use of digital devices makes your parental role easier, neither easier nor more difficult, or more difficult.	Open-ended response
3	Have you ever discussed your child’s use of digital devices with a pediatrician, child neuropsychiatrist, or other healthcare professional?	Yes/No
4	Are you aware that guidelines on children’s use of digital devices are available?	Yes/No
5	Do you use timers or auto-lock functions (e.g., automatic shutdown) to limit your child’s use of electronic devices?	Yes/No
6	Do you use parental controls or other tools to restrict your child’s access to specific digital content?	Yes/No

**Table 2:** Demographic and clinical characteristics of the sample inserted as variables in the analysis.

	*n*	%
Responding parent		
Mother	292	83
Father	58	16
Parents’ age (years)		
≤45	183	52
>45	169	48
Socioeconomic status		
Low/middle	77	22
Middle-high	180	51
High	95	27
Children with NDs		
One	302	86
More than one	50	14
Functional disability level (highest among siblings)		
Mild	171	49
Moderate	102	29
Severe-to-complete	79	22

**Table 3:** Parents’ perspective, awareness, and practices on child screen time. Answers are reported as *n* (%).

	Yes		Neutral	No	
	**Very Much**	**Somewhat Yes**		**Somewhat No**	**Not At All**
Benefits for child development	37 (11)	100 (28)	91 (26)	107 (30)	17 (5)
Support for parenting	17 (5)	76 (21)	112 (32)	84 (24)	63 (18)
	**Yes**			**No**	
Discussed with health professionals	177 (50)			175 (50)	
Aware of guidelines	243 (69)			109 (31)	
Time-limiting tools	124 (35)			228 (65)	
Content-limiting tools	180 (51)			172 (49)	

**Table 4:** Parents’ perspective about child screen time according to demographic and clinical characteristics of the sample. Percentages of positive responses are reported. Significant comparisons (*p* < 0.05) are highlighted in bold.

	Answer “Yes” (%)					
	Benefits for child development	Support for parenting	Discussed with health professionals	Aware of guidelines	Time-limiting tools	Content-limiting tools
Responding parent						
Mother	**36**	**23**	50	70	35	52
Father	**55**	**43**	50	67	36	50
Parents’ age (years)						
≤45	34	23	48	72	**40**	53
>45	44	30	53	66	**30**	49
Socioeconomic status						
Low/middle	39	29	45	61	34	55
Middle-high	42	25	52	72	31	50
High	34	27	52	71	45	50
Children with NDs						
One	40	27	49	67	34	50
More than one	35	24	57	78	43	55
Functional disability						
Mild	**26**	**20**	**43**	72	**34**	56
Moderate	**51**	**29**	**56**	65	**45**	53
Severe-to-complete	**52**	**37**	**58**	68	**25**	39

**Table 5:** Themes and subthemes identified in parents’ responses about the effects of digital media use on their parental role.

Theme	Subtheme	*n*	Examples
**Educational means**			
	Communication	13	“It helps me communicate with my nonverbal daughter by using specific programs”
	Learning	42	“My child often uses devices as support for doing homework”
	Socialisation	9	“Because I’ve always seen it as a way to interact with others”
	Activities for parents	8	“It helps me suggest activities, such as singing or listening to audiobooks”
**Entertainment**			
	Digital pacifier/babysitter	33	“I can get other things done at home while he watches cartoons”
	Leisure time	26	“We use digital devices for watching cartoons, which often serve as a relaxing activity for my child”
**Reward**		3	“It’s a reward for managing his oppositional/provocative behaviour”
**Screen time as a “necessity”**		11	“Technology is now part of our daily routine and has its own place at the right moments”
**Negative effects on the child**			
	Detachment from reality	45	“My son becomes absent, detached from the real world”
	Socialisation	15	“Because there is less dialogue and fewer opportunities to do things together”
	Mood and behaviour	19	“The child becomes more aggressive when using devices”
**Parental behaviour and attitudes**			
	Parental control and authority	16	“The parent loses authority”
	Beliefs and values	39	“They fill children’s heads with useless or false information”
	“Just a tool”	29	“It is just a tool that should be used consciously and under supervision”

## Data Availability

The data supporting this study are available upon reasonable request by contacting the corresponding author by e-mail.

## References

[1] Straub L., Bateman B.T., Hernandez-Diaz S., York C., Lester B., Wisner K.L., McDougle C.J., Pennell P.B., Gray K.J., Zhu Y. (2022). Neurodevelopmental Disorders Among Publicly or Privately Insured Children in the United States. JAMA Psychiatry.

[2] Francés L., Quintero J., Fernández A., Ruiz A., Caules J., Fillon G., Hervás A., Soler C.V. (2022). Current State of Knowledge on the Prevalence of Neurodevelopmental Disorders in Childhood According to the DSM-5: A Systematic Review in Accordance with the PRISMA Criteria. Child Adolesc. Psychiatry Ment. Health.

[3] Chadwick D., Ågren K.A., Caton S., Chiner E., Danker J., Gómez-Puerta M., Heitplatz V., Johansson S., Normand C.L., Murphy E. (2022). Digital Inclusion and Participation of People with Intellectual Disabilities during COVID-19: A Rapid Review and International Bricolage. J. Policy Pract. Intellect. Disabil..

[4] Ophir Y., Rosenberg H., Tikochinski R., Dalyot S., Lipshits-Braziler Y. (2023). Screen Time and Autism Spectrum Disorder. JAMA Netw. Open.

[5] Kwon S., Armstrong B., Wetoska N., Capan S. (2024). Screen Time, Sociodemographic Factors, and Psychological Well-Being among Young Children. JAMA Netw. Open.

[6] Santos R.M.S., Mendes C.G., Sen Bressani G.Y., de Alcantara Ventura S., de Almeida Nogueira Y.J., de Miranda D.M., Romano-Silva M.A. (2023). The Associations between Screen Time and Mental Health in Adolescents: A Systematic Review. BMC Psychol..

[7] Yuan G., Zhu Z., Guo H., Yang H., Zhang J., Zhang K., Zhang X., Lu X., Du J., Shi H. (2024). Screen Time and Autism Spectrum Disorder: A Comprehensive Systematic Review of Risk, Usage, and Addiction. J. Autism Dev. Disord..

[8] Mazurek M.O., Shattuck P.T., Wagner M., Cooper B.P. (2012). Prevalence and Correlates of Screen-Based Media Use among Youths with Autism Spectrum Disorders. J. Autism Dev. Disord..

[9] Xue Y., Tian P., Zhang Y., Bai M.S., Jia F.Y. (2026). Impact of Screen Time on White Matter Integrity and Neurodevelopment in Children with Autism Spectrum Disorder: A Diffusional Kurtosis Imaging Mediation Analysis. Eur. Child Adolesc. Psychiatry.

[10] Takahashi N., Tsuchiya K.J., Okumura A., Harada T., Iwabuchi T., Rahman M.S., Kuwabara H., Nomura Y., Nishimura T. (2023). The Association between Screen Time and Genetic Risks for Neurodevelopmental Disorders in Children. Psychiatry Res..

[11] World Health Organization (2019). Guidelines on Physical Activity, Sedentary Behaviour and Sleep for Children under 5 Years of Age. WHO Guidelines Approved by the Guidelines Review Committee.

[12] Council on Communications and Media (2016). Media and Young Minds. Pediatrics.

[13] Bozzola E., Spina G., Ruggiero M., Memo L., Agostiniani R., Bozzola M., Corsello G., Villani A. (2018). Media Devices in Pre-School Children: The Recommendations of the Italian Pediatric Society. Ital. J. Pediatr..

[14] Bozzola E., Spina G., Ruggiero M., Vecchio D., Caruso C., Bozzola M., Staiano A.M., Agostiniani R., Del Vecchio A., Banderali G. (2019). Media Use during Adolescence: The Recommendations of the Italian Pediatric Society. Ital. J. Pediatr..

[15] Bozzola E., Irrera M., Barni S., Caruso C., Leccese B., Franzese E., Fioretti L., Bernardelli L., Scarpato E., Cupertino V. (2026). Digital Media Exposure and Pediatric Health: The Recommendations from the Italian Society of Pediatrics Digital Dependency Commission. Ital. J. Pediatr..

[16] Mayer Y., Cohen-Eilig M., Chan J., Kuzyk N., Glodjo A., Jarus T. (2024). Digital Citizenship of Children and Youth with Autism: Developing Guidelines and Strategies for Caregivers and Clinicians to Support Healthy Use of Screens. Autism.

[17] Mayer Y., Nguyen K., Lei E., Cohen-Eilig M., Glodjo A., Jarus T. (2025). Enhancing Digital Citizenship of Children and Youth with Autism: Evaluating Novel Screen Time Guidelines for Caregivers and Professionals. Child. Youth Serv. Rev..

[18] McArthur B.A., Volkova V., Tomopoulos S., Madigan S. (2022). Global Prevalence of Meeting Screen Time Guidelines among Children 5 Years and Younger: A Systematic Review and Meta-Analysis. JAMA Pediatr..

[19] Butti N., Mascheroni E., Masserano F., Nossa R., Cordolcini L., Riva B., Biffi E., Montirosso R. (2026). Screen Time in Children with Neurodevelopmental Disorders and Their Parents: A Survey-Based Study in a Paediatric Italian Sample. Disabil. Rehabil. Assist. Technol..

[20] Smith E.M., Huff S., Wescott H., Daniel R., Ebuenyi I.D., O’Donnell J., Maalim M., Zhang W., Khasnabis C., MacLachlan M. (2024). Assistive Technologies Are Central to the Realization of the Convention on the Rights of Persons with Disabilities. Disabil. Rehabil. Assist. Technol..

[21] Pontikas C.M., Tsoukalas E., Serdari A. (2022). A Map of Assistive Technology Educative Instruments in Neurodevelopmental Disorders. Disabil. Rehabil. Assist. Technol..

[22] Lin J., Magiati I., Chiong S.H.R., Singhal S., Riard N., Ng I.H.X., Muller-Riemenschneider F., Wong C.M. (2019). The Relationship among Screen Use, Sleep, and Emotional/Behavioral Difficulties in Preschool Children with Neurodevelopmental Disorders. J. Dev. Behav. Pediatr..

[23] Dong H.Y., Wang B., Li H.H., Yue X.J., Jia F.Y. (2021). Correlation Between Screen Time and Autistic Symptoms as Well as Development Quotients in Children with Autism Spectrum Disorder. Front. Psychiatry.

[24] Bronfenbrenner U. (1979). The Ecology of Human Development: Experiments by Design and Nature.

[25] Munzer T., Milkovich L.M., Madigan S., Tomopoulos S., Parga-Belinkie J., Ajumobi T., Cross C., Gerwin R., Radesky J., Evans Y. (2026). Digital Ecosystems, Children, and Adolescents: Technical Report. Pediatrics.

[26] Munzer T., Parga-Belinkie J., Milkovich L.M., Tomopoulos S., Ajumobi T., Cross C., Gerwin R., Madigan S., Psych R., Radesky J. (2026). Digital Ecosystems, Children, and Adolescents: Policy Statement. Pediatrics.

[27] Mascheroni G., Zaffaroni L.G. (2023). From “Screen Time” to Screen Times: Measuring the Temporality of Media Use in the Messy Reality of Family Life. Communications.

[28] Navarro J.L., Fletcher A., Jensen M. (2023). A Bifactor Model of U.S. Parents’ Attitudes Regarding Mediation for the Digital Age. J. Child. Media.

[29] Coyne S.M., Radesky J., Collier K.M., Gentile D.A., Linder J.R., Nathanson A.I., Rasmussen E.E., Reich S.M., Rogers J. (2017). Parenting and Digital Media. Pediatrics.

[30] Altindağ Kumaş Ö., Sardohan Yildirim A.E. (2024). Exploring Digital Parenting Awareness, Self-Efficacy and Attitudes in Families with Special Needs Children. Br. J. Educ. Technol..

[31] Gurgor-Kilic F.G., Tunc-Paftali A., Yildiz G. (2025). Views of Parents of Children with Neurodevelopmental Disabilities on Their Children’s Social Media Use and Media Parenting. Int. J. Dev. Disabil..

[32] Lane R., Radesky J. (2019). Digital Media and Autism Spectrum Disorders: Review of Evidence, Theoretical Concerns, and Opportunities for Intervention. J. Dev. Behav. Pediatr..

[33] Alfredsson Ågren K., Kjellberg A., Hemmingsson H. (2020). Internet Opportunities and Risks for Adolescents with Intellectual Disabilities: A Comparative Study of Parents’ Perceptions. Scand. J. Occup. Ther..

[34] Molin M., Sorbring E., Löfgren-Mårtenson L. (2015). Teachers’ and Parents’ Views on the Internet and Social Media Usage by Pupils with Intellectual Disabilities. J. Intellect. Disabil..

[35] Geurts S.M., Koning I.M., Vossen H.G.M., van den Eijnden R.J.J.M. (2022). Rules, Role Models or Overall Climate at Home? Relative Associations of Different Family Aspects with Adolescents’ Problematic Social Media Use. Compr. Psychiatry.

[36] Chiner E., Gómez-Puerta M., Cardona-Moltó M.C. (2017). Internet and People with Intellectual Disability: An Approach to Caregivers’ Concerns, Prevention Strategies and Training Needs. J. New Approaches Educ. Res..

[37] Elias N., Sulkin I. (2019). Screen-Assisted Parenting: The Relationship Between Toddlers’ Screen Time and Parents’ Use of Media as a Parenting Tool. J. Fam. Issues.

[38] Pardo-Salamanca A., Gómez S., Santamarina C., Pastor G., Berenguer C. (2025). The Effectiveness of an Online-Based Psychosocial Program for Parents of Children with Neurodevelopmental Disorders—A Randomized Controlled Trial. Child Adolesc. Ment. Health.

[39] Tan-MacNeill K.M., Smith I.M., Johnson S.A., Chorney J., Corkum P. (2021). A Systematic Review of Online Parent-Implemented Interventions for Children with Neurodevelopmental Disorders. Child. Health Care.

[40] Barr R., Kirkorian H., Radesky J., Coyne S., Nichols D., Blanchfield O., Rusnak S., Stockdale L., Ribner A., Durnez J. (2020). Beyond Screen Time: A Synergistic Approach to a More Comprehensive Assessment of Family Media Exposure During Early Childhood. Front. Psychol..

[41] Domoff S.E., Harrison K., Gearhardt A.N., Gentile D.A., Lumeng J.C., Miller A.L. (2019). Development and Validation of the Problematic Media Use Measure: A Parent Report Measure of Screen Media “Addiction” in Children. Psychol. Pop. Media Cult..

[42] Klakk H., Wester C.T., Olesen L.G., Rasmussen M.G., Kristensen P.L., Pedersen J., Grøntved A. (2020). The Development of a Questionnaire to Assess Leisure Time Screen-Based Media Use and Its Proximal Correlates in Children (SCREENS-Q). BMC Public Health.

[43] Coutinho F., Saxena G., Shah A., Tilak S., Desai N., Udani V. (2022). Mobile Media Exposure and Use in Children Aged Zero to Five Years with Diagnosed Neurodevelopmental Disability. Disabil. Rehabil. Assist. Technol..

[44] Suresh A., Tiwari S. (2023). Parental Knowledge, Attitudes and Concerns towards Media Technology and Screen Time Use in Children with ASD and Typically Developing Children. Technol. Disabil..

[45] Mallawaarachchi S.R., Hooley M., Sutherland-Smith W., Horwood S. (2022). “You’re Damned If You Do, You’re Damned If You Don’t”: A Qualitative Exploration of Parent Motives for Provision of Mobile Screen Devices in Early Childhood. BMC Public Health.

[46] Galvan M.J., Payne B.K., Hannay J., Georgeson A.R., Muscatell K.A. (2023). What Does the MacArthur Scale of Subjective Social Status Measure? Separating Economic Circumstances and Social Status to Predict Health. Ann. Behav. Med..

[47] Perneger T.V. (1998). What’s Wrong with Bonferroni Adjustments. BMJ.

[48] Parker R.A., Weir C.J. (2020). Non-Adjustment for Multiple Testing in Multi-Arm Trials of Distinct Treatments: Rationale and Justification. Clin. Trials.

[49] Braun V., Clarke V. (2006). Using Thematic Analysis in Psychology. Qual. Res. Psychol..

[50] Braun V., Clarke V. (2019). Reflecting on Reflexive Thematic Analysis. Qual. Res. Sport. Exerc. Health.

[51] Fereday J., Muir-Cochrane E. (2006). Demonstrating Rigor Using Thematic Analysis: A Hybrid Approach of Inductive and Deductive Coding and Theme Development. Int. J. Qual. Methods.

[52] Xu W., Zammit K. (2020). Applying Thematic Analysis to Education: A Hybrid Approach to Interpreting Data in Practitioner Research. Int. J. Qual. Methods.

[53] Chong S.C., Teo W.Z., Shorey S. (2023). Exploring the Perception of Parents on Children’s Screentime: A Systematic Review and Meta-Synthesis of Qualitative Studies. Pediatr. Res..

[54] Braun V., Clarke V. (2016). (Mis)Conceptualising Themes, Thematic Analysis, and Other Problems with Fugard and Potts’ (2015) Sample-Size Tool for Thematic Analysis. Int. J. Soc. Res. Methodol..

[55] Naeem M., Ozuem W., Howell K., Ranfagni S. (2024). Demystification and Actualisation of Data Saturation in Qualitative Research Through Thematic Analysis. Int. J. Qual. Methods.

[56] Borgström Å., Daneback K., Molin M. (2019). Young People with Intellectual Disabilities and Social Media: A Literature Review and Thematic Analysis. Scand. J. Disabil. Res..

[57] Livingstone S., Blum-Ross A. (2020). Parenting for a Digital Future: How Hopes and Fears About Technology Shape Children’s Lives.

[58] Zorzi S., Lubkina V., Jēkabsone I., Berteotti L. (2025). Digital Technology Interventions for Communication Skills in Persons with Neurodevelopmental Disorders: A Scoping Review. Res. Dev. Disabil..

[59] Shandra C.L. (2021). Disability and Patterns of Leisure Participation Across the Life Course. J. Gerontol. Ser. B.

[60] Tang L., Hruska V., Ma D.W.L., Haines J. (2021). Parenting under Pressure: Stress Is Associated with Mothers’ and Fathers’ Media Parenting Practices in Canada. J. Child. Media.

[61] Kattein E., Schmidt H., Witt S., Jörren H.L., Menrath I., Rumpf H.J., Wartberg L., Pawils S. (2023). Increased Digital Media Use in Preschool Children: Exploring the Links with Parental Stress and Their Problematic Media Use. Children.

[62] Craig F., Operto F.F., De Giacomo A., Margari L., Frolli A., Conson M., Ivagnes S., Monaco M., Margari F. (2016). Parenting Stress among Parents of Children with Neurodevelopmental Disorders. Psychiatry Res..

[63] Tang L., Darlington G., Ma D.W.L., Haines J. (2018). Mothers’ and Fathers’ Media Parenting Practices Associated with Young Children’s Screen-Time: A Cross-Sectional Study. BMC Obes..

[64] Hinkley T., McCann J.R. (2018). Mothers’ and Father’s Perceptions of the Risks and Benefits of Screen Time and Physical Activity during Early Childhood: A Qualitative Study. BMC Public Health.

[65] Mollborn S., Limburg A., Pace J., Fomby P. (2022). Family Socioeconomic Status and Children’s Screen Time. J. Marriage Fam..

[66] Sorbring E., Molin M., Löfgren-Mårtenson L. (2017). “I’m a Mother, but I’m Also a Facilitator in Her Every-Day Life”: Parents’ Voices about Barriers and Support for Internet Participation among Young People with Intellectual Disabilities. Cyberpsychol. J. Psychosoc. Res. Cyberspace.

[67] Bergmann C., Dimitrova N., Alaslani K., Almohammadi A., Alroqi H., Aussems S., Barokova M., Davies C., Gonzalez-Gomez N., Gibson S.P. (2022). Young Children’s Screen Time during the First COVID-19 Lockdown in 12 Countries. Sci. Rep..

[68] Ke Y., Chen S., Hong J., Liang Y., Liu Y. (2023). Associations between Socioeconomic Status and Screen Time among Children and Adolescents in China: A Cross-Sectional Study. PLoS ONE.

[69] Fernández-Batanero J.M., Montenegro-Rueda M., Fernández-Cerero J., García-Martínez I. (2022). Assistive Technology for the Inclusion of Students with Disabilities: A Systematic Review. Educ. Technol. Res. Dev..

[70] Osorio-Saez E.M., Eryilmaz N., Sandoval-Hernandez A. (2021). Parents’ Acceptance of Educational Technology: Lessons from Around the World. Front. Psychol..

[71] Reid Chassiakos Y.L., Radesky J., Christakis D., Moreno M.A., Cross C., Hill D., Ameenuddin N., Hutchinson J., Levine A., Boyd R. (2016). Children and Adolescents and Digital Media. Pediatrics.

[72] Beyens I., Eggermont S. (2014). Putting Young Children in Front of the Television: Antecedents and Outcomes of Parents’ Use of Television as a Babysitter. Commun. Q..

[73] Milford S.C., Vernon L., Scott J.J., Johnson N.F. (2025). Exploring Parent Self-Efficacy in Children’s Digital Device Use: Understanding Shame and Self-Stigma through a Mixed-Methods Approach. Int. J. Child-Comput. Interact..

[74] Gillespie-Smith K., Hendry G., Anduuru N., Laird T., Ballantyne C. (2021). Using Social Media to Be ‘Social’: Perceptions of Social Media Benefits and Risk by Autistic Young People, and Parents. Res. Dev. Disabil..

[75] Seale J. (2014). The Role of Supporters in Facilitating the Use of Technologies by Adolescents and Adults with Learning Disabilities: A Place for Positive Risk-Taking?. Eur. J. Spec. Needs Educ..

[76] Heitplatz V.N., Bühler C., Hastall M.R. (2022). Usage of Digital Media by People with Intellectual Disabilities: Contrasting Individuals’ and Formal Caregivers’ Perspectives. J. Intellect. Disabil..

